# Protein Functional Surfaces: Global Shape Matching and Local Spatial Alignments of Ligand Binding Sites

**DOI:** 10.1186/1472-6807-8-45

**Published:** 2008-10-27

**Authors:** T Andrew Binkowski, Andrzej Joachimiak

**Affiliations:** 1 Midwest Center for Structural Genomics and Structural Biology Center, Biosciences Division, Argonne National Laboratory, Argonne, Illinois, 60439, USA

## Abstract

**Background:**

Protein surfaces comprise only a fraction of the total residues but are the most conserved functional features of proteins. Surfaces performing identical functions are found in proteins absent of any sequence or fold similarity. While biochemical activity can be attributed to a few key residues, the broader surrounding environment plays an equally important role.

**Results:**

We describe a methodology that attempts to optimize two components, global shape and local physicochemical texture, for evaluating the similarity between a pair of surfaces. Surface shape similarity is assessed using a three-dimensional object recognition algorithm and physicochemical texture similarity is assessed through a spatial alignment of conserved residues between the surfaces. The comparisons are used in tandem to efficiently search the Global Protein Surface Survey (GPSS), a library of annotated surfaces derived from structures in the PDB, for studying evolutionary relationships and uncovering novel similarities between proteins.

**Conclusion:**

We provide an assessment of our method using library retrieval experiments for identifying functionally homologous surfaces binding different ligands, functionally diverse surfaces binding the same ligand, and binding surfaces of ubiquitous and conformationally flexible ligands. Results using surface similarity to predict function for proteins of unknown function are reported. Additionally, an automated analysis of the ATP binding surface landscape is presented to provide insight into the correlation between surface similarity and function for structures in the PDB and for the subset of protein kinases.

## Background

It has become apparent that surfaces, comprised of a fraction of the total residues, are the most conserved functional features of proteins. Proteins utilize common surface motifs to create precise chemical environments designed to perform specific functions. These motifs are not restricted to a single protein scaffold but can be found within different protein folds or at domain/domain and subunits interfaces. While biochemical activity can be attributed to a few key residues (e.g catalytic triads), the broader surrounding environment (i.e. auxiliary residues in spatial proximity) often plays an equally import role in fine-tuning molecular recognition and/or catalysis.

Powerful evolutionary forces have allowed proteins to govern ligand binding through seemingly subtle local surface variability. These changes, which are not easily detectable by sequence analysis, may provide competitive advantage for optimization of co-factor specificity. In some circumstances, surface diversity adversely affects normal cell process by providing environments for undesired binding events (e.g. drug side effects) or mutations directly correlated to disease[1]. The conservation of functional surfaces presents an opportunity to compare and analyze proteins independent of sequence or fold. These comparisons can be used to classify protein functions or to infer biochemical activity for proteins with unknown function, such as those targeted by structural genomics programs.

Several methods have been developed detecting localized, spatial protein similarities with applications for evolutionary analysis, function prediction and drug discovery. The use of graph theory has been widely applied to the comparison of three-dimensional patterns. Artymiuk *et al*. developed an algorithm based on subgraph isomorphism detection to search residue patterns against the PDB[2]. Kinoshita *et al*. used clique detection algorithms to assign protein biochemical functions using the similarity information of molecular surface geometries and electrostatic potentials[3]. Using a clique-detection algorithm, Schmitt *et al*., compared generic pseudo-centers that code for possible ligand-protein interactions in protein cavities. Query cavities are searched against Cavbase, a pre-computed database of cavities extracted from the PDB[4]. The method has been applied to identify surfaces in non-homologous proteins as well as for the classification of protein families[5]. Kleywegt searched for motifs of residue pseudo-centers in a library of protein structures using a depth-first search algorithm[6]. Russell also developed an algorithm based on depth-first search that detects atomic geometric patterns common in between side-chains in proteins and presented new examples of convergent evolution[7]. Parametric statistical evaluations of Russell's atomic superposition method were extended by Stark *et al*. [8].

Another widely used approach is geometric hashing, which is an efficient method for matching features against a database. Jackson and Gold used geometric hashing to perform an all-against-all comparison of protein-ligand binding sites in the SitesBase database [9-11]. Their method was also applied for functional annotation and building pharmacophore models for drug discovery[11]. Fischer *et. al*. developed an algorithm based on geometric hashing that detects surface similarities of proteins using spatial patterns of atoms[12,13]. A similar method, TESS, has been applied for the derivation and matching of annotated spatial templates[14]. JESS[15], a successor to TESS, searches small groups of atoms under arbitrary constraints on geometry and chemistry and utilized statistics to evaluate matches. It is used to query the Catalytic Site Atlas (CSA)[16] a collection of annotated residues patterns extracted from manual literature searches. JESS is also used in the PROFUNC[17] suite of annotation tools in the reverse template search, where a radius defined perimeter extends a local residue pattern search for improved search specificity.

A protein evolution based method, pvSOAR, was developed that used the unique approach of aligning sub-sequences of surface residues to establish a residue correspondence between surfaces[18,19]. The residues were then superimposed on each other and statistical significance was evaluated for the resulting RMSD. This method was used to detect similar functional surfaces in non-homologous proteins. Furthermore, in a recent study of shape variation of ligand binding pockets, Kahraman *et. al*., used a shape-only comparison metric based on spherical harmonics[20]. It was shown that shape descriptors could be used to classify ligand into their binding sites.

In this study, we describe a new method for the sequence order independent comparison and alignment of protein functional surfaces. Our method, *SurfaceScreen*, attempts to optimize two components, global surface shape and local physicochemical texture, for evaluating the similarity between a pair of surfaces. Surface shape similarity is assessed using a three-dimensional object recognition algorithm and is used to rapidly pre-classify surfaces from a large library of surfaces. Surfaces with sufficient shape complimentarity are then aligned by combinatorially identifying the best superimposition of common residues between the two surfaces. We introduce several metrics for scoring different properties of a surface alignment and an overall scoring function used in library searches. Furthermore, we introduce the Global Protein Surface Survey (GPSS), a library of annotated protein surfaces calculated from all structures in the PDB. Querying surfaces from proteins of unknown function against the GPSS library allows *SurfaceScreen *to be utilized as predictive tool.

We describe three types of analysis to assess surface shape comparisons and spatial alignments. First, we describe the retrieval of surfaces from the GPSS library for surfaces, from the same protein, that bind ligands of various size, shape and pharmacophore properties. For this we use the example of HIV-1 protease. Second, we use the example of heme (iron-protoporphyrin IX) binding sites to describe the retrieval of a functionally diverse binding surface that binds the same ligand. We provide the example of using our method as an annotation tool, identifying a new member of the heme binding monooxygenase family. Third, we describe how conformational diversity of bound ligands impacts retrieval rate for ubiquitous nucleotide binding sites. We also present the example of a nucleotide binding surface prediction and crystallographic validation for a structural genomics target with a new fold. We conclude with an analysis of the ATP binding surface landscape to provide insight on the correlation between surface similarity and function for structures in the PDB and for the subset of protein kinases.

## Methods

### Theory and Algorithms

While key conserved residues are localized within a surface for function, additional residues contribute to the overall size and shape of the functional surface environment. In some instances, these non-key residues play an important, but non-obvious, functional role. This has been shown for aminopeptidases, where the mouth opening diameter filters peptide access into the active sites[21,22], or for N-acetyltransferases, where the acetyl-CoA and substrate binding surfaces are sub-pockets within a larger cleft[23]. In other instances, these auxiliary residues have no obvious relevance, being positioned simply as a result of folding and structural requirements, codon pressure or unrelated functional specifications. Therefore, a detachment exists between global and local surface properties of functional sites, which can distort comparison measures and mask similarities.

Our approach to identifying meaningful similarities between two surfaces is accomplished by decomposing surface comparisons into two components, global shape and local physicochemical texture. Global shape comparisons can be a very powerful precursor of overall surface similarity. They are computed very efficiently and can help rapidly reject grossly dissimilar surfaces. This is followed by a non-heuristic spatial residue alignment, which assures the optimal superposition of conserved residues between two surfaces.

#### Surface Shape Signatures for Rapid Global Surface Shape Comparison

Unlike protein folds, a common lexicon has not emerged to precisely describe the shapes formed by surfaces yet quantitative measures can be computed to empirically describe shape. Here, we introduce a metric, the *SurfaceShapeSignature *(*SSS*), which describes the global shape of a protein surface that can be used for comparison. Adapted from three-dimensional database object retrieval, the method represents the signature of an object as a probability distribution sampled from a shape function measuring global geometric properties[24]. The complexity of shape matching is then reduced to the comparison of two probability distributions. This approach was used to quickly and successfully retrieve and classify complex shapes from three-dimensional databases.

**Figure 1:**
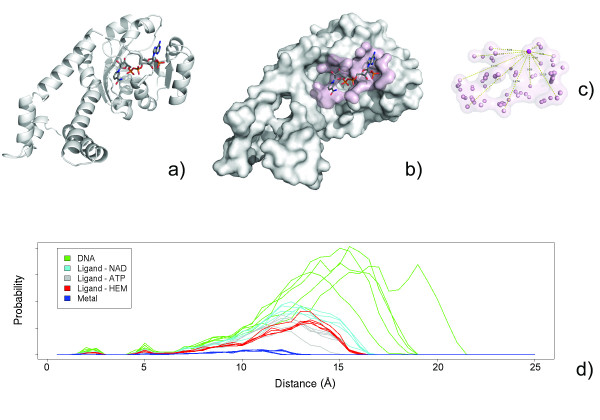
**Automated identification of protein binding surfaces and construction of *SurfaceShapeSignatures *(*SSS*)**. The nicotinamide-adenine-dinucleotide phosphate (NADP) binding surface from human pathogen *S. pyogenes *(PDB:2ahr, a) is defined by measuring the change in solvent accessibility between the bound and apo structure (b, pink). The *SSS *of a binding surface is constructed by measuring the inter-atomic Euclidean distances between all unique surface atom pairs (c). The signatures of select DNA, ligand and metal binding surfaces for proteins in the PDB.

After a protein's binding surfaces has been identified (see Methods), the *SSS *is constructed by systematically measuring the Euclidean distance between all unique atom pairs for a given surface. This is seen for the nicotinamide-adenine-dinucleotide phosphate (NADP) binding surface from human pathogen *S. pyogenes *(PDB:2ahr) in Figure 1c. The inter-atomic distances are then sorted to form the shape signatures. The *SSS *distributions for a selection of heme, nicotinamide adenine dinucleotide (NAD) and adenosine 5'-triphosphate (ATP) binding surfaces are shown in Figure 1d. For reference, *SSS *distributions for a selection of DNA and metal binding surfaces are also shown. Once the shape distributions for two surfaces are computed, we apply the Kolmogorov-Smirnov (KS) test[25] to compare the probability distributions. The KS test identifies the greatest distance between the observed and expected cumulative frequencies and is bound between zero (identical distributions) and 1 (different distributions).

#### Advantages and Limitations of Surface Shape Signatures

The primary advantages of this approach are computational robustness and efficiency. In the original work of Osada, the robustness of shape signatures was verified against a variety of transformations including scale, rotation and mirroring and were insensitive to model simplification. These properties are ideal for applications in structural biology as minor conformational changes or small surface perturbations should not mask overall shape similarities. For example, we should be able to detect the similarity between the apo and bound states of a binding site, yet still discern between the two states. Insensitivity to model simplification allows for meaningful comparisons between surfaces comprised of non-trivial atoms count differences. The implementation and execution of this algorithm is computationally straightforward and allows a query surface to be searched against the GPSS library in minutes.

Biochemical functions rely on a combination of shape and chemical compatibility and therefore one should not expect that shape alone could describe this complexity. Figure 1 shows that NAD, ATP, and heme binding surfaces share similar and, in some cases, overlapping distributions. The *SSS *comparison can convey gross shape similarity but is void of chemical typing information and cannot be used to infer specific functional information. Instead, it provides a fast and robust comparison metric utilized as the entry point into more comprehensive surface shape matching methodology.

#### Surface Shape Signature Similarity Threshold

**Figure 2:**
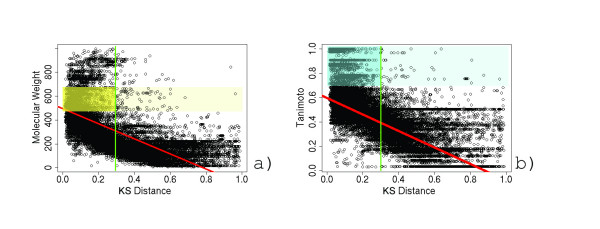
**Identification of a threshold for *SurfaceShapeSignatures***. *SSS *distances obtained by querying the ATP binding site of cAMP-dependent kinase (PDB:1atp) against the GPSS ligand surface library are plotted against the molecular weight of the ligand corresponding to the library surface (a). Ligands with MW ± 100 D of ATP are highlighted in yellow. The molecular shape similarity Taniomoto score between ATP and the ligand corresponding to the library surface is plotted in (b). Tanimoto scores greater than 0.7 (blue) are generally regarded as similar. The correlation coefficients for molecular weight and shape similarity are 0.46 and 0.45, respectively, and the corresponding regression lines are shown in red. Our selected threshold distance of 0.3 (green) for use in our *SurfaceScreen *methodology eliminates less than 1% of true-positive surfaces in our benchmarking exercises.

The choice of a shape similarity threshold has a significant impact on the efficiency and specificity of surface library searching. Operating under the pretense that ligands with similar shape and molecular weight are more likely to bind to similar pockets, we correlated these properties to *SSS *KS distances. Our training data is taken from querying cAMP-dependent kinase (PDB:1atp) ATP binding surface against the GPSS library. With a correlation coefficient of 0.458, we see that some degree of surface similarity can be inferred simply by molecular weight (Figure 2a). We highlight a region on the plot (yellow) that corresponds to +/- 100 D from the molecular weight of ATP and identify the similarity distance of 0.22, along the x-axis, in which only 11% of surfaces exist as outliers. Next, ATP was compared to ligands corresponding to the surfaces in our library using the molecular shape matching application ROCS (OpenEye Scientific Software, Inc). ROCS identifies the best superposition of two molecules by optimizing their overlapping volume and reports a normalized Tanimoto score. Tanimoto values greater than 0.7 are generally regarded as having similar shape and are highlighted on our plot (cyan). The Tanimoto scores are correlated to the SSS distance in Figure 2b. We identify a distance of 0.24, in which only 10% are outliers. Since our assumptions about molecular weight and ligand shape similarity are simplistic and surface comparison hope to identify more evolutionary distant relationships, we set our default threshold distance at 0.3. In the benchmarking retrieval experiments presented in Results section, our default threshold excludes less than 1% of true-positive surfaces, all of which can be justified by unique structural incident (e.g. multiple binding pockets, mutation experiments, low resolution structure, crystallographic error).

#### Local Spatial Surface Residue Alignments for Physicochemical Texture Similarity

The spatial arrangement of localized surface patterns and orientation of side chains are used to assess our evaluations of biochemical function complimentarity. An exhaustive enumeration and search algorithm, *SurfaceAlign*, is applied to detect groups of spatially conserved amino acid residue sets. In this application, the term "conserved" refers to the identical residues common to two protein surfaces. Our three-dimensional representation of a surface residue is represented by a single point located at the center of mass of all atoms identified as contributing to a particular surface.

**Figure 3:**
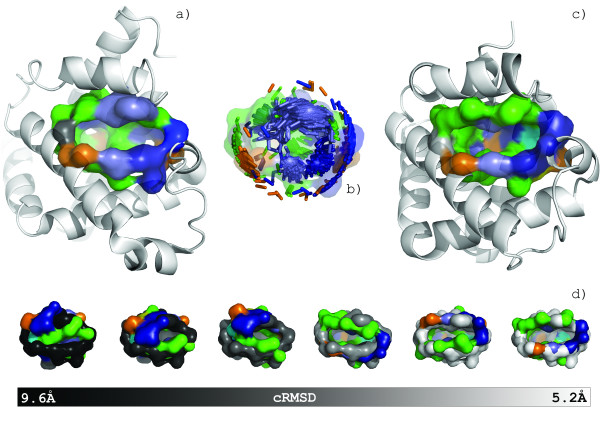
**The *SurfaceAlign *algorithm identifies the optimal alignment of spatially conserved residues**. *6,220,800 *alignment combinations and permutations are required for the alignment of 25 conserved residues of the heme binding pockets of myoglobin from *P. catodon *(a) and structural genomics target hemoglobin alpha-1 from *P. flavescens *(c). 100 alignment solutions are shown in stick representations (b). An alignment series shows the superposition of the solutions calculated towards converging to the optimal alignment (d). The myobglobin query surface is shaded in grayscale to represent the cRMSD values (black represents a large cRMSD and white represent small cRMSD) and the hemoglobin surface is colored by the shapely color scheme[77].

The alignment of two surfaces is performed by combinatorially identifying the best superposition of the maximum subset of conserved residues between surfaces. For all conserved residues of each type, we enumerate all combinations and permutations to create unique coordinate sets of common residues between the two surfaces. A visual depiction of an alignment between the heme binding pockets of myoglobin from *P. catodon *(PDB:1mbn) and structural genomics target hemoglobin alpha-1 (PDB:1xq5) is shown in Figure 3. The geometric dissimilarity of each coordinate combination is evaluated following the methods of Umeyama[26], which is based on singular value decomposition of the correlation matrix of the coordinates to identify the least square rotational matrix, translation vector and the root mean square distance (RMSD). We utilize two variants of the RMSD: the coordinate root mean square distance (cRMSD), for atomic coordinates as represented in our residue model, and the orientation root mean square distance (oRMSD)[18]. The oRMSD is a derivative dissimilarity measure that reduces the effect of outliers on an RMSD value and simulates the conformational flexibility of amino acid side chains.

#### Statistical Significance of Aligned Surface Distances

RMSD calculations are sensitive to the number of data points compared, making it necessary to assess the statistical significance of raw distance measures. This is accomplished by converting calculated cRMSD and oRMSD values to a probability value (P-value) measuring the likelihood of obtaining a specific RMSD value for a solution with a given number of residues. This allows for the meaningful comparisons between alignments with differing number of common residues. Following the method described by Binkowski *et al*[18], random surface alignments were performed for alignment solutions of *N*_*res *_residues, where 3 ≤ *N*_*res *_≤ 100. Calculations numbering 10^10 ^were computed to construct lookup tables associating cRMSD and oRMSD scores to P-values. To minimize the inherent bias in the PDB toward particular protein families, special consideration was employed to utilize a non-redundant surface library consisting of proteins sharing less than 90% whole-protein sequence similarity.

#### Surface Volumes Overlap of Aligned Surfaces

While the alignment compares key residues important for shared biochemical function, the volume overlap of an alignment provides a comparison of all atoms belonging to a surface. The overlap volume is defined as the volume difference between the superimposed surfaces and is calculated from the formula:

*V*_*AB *_= *V*_*A *_+ *V*_*B *_- *V*_*A*∪*B*_

Where *V*_*A*_, *V*_*B*_, and *V*_*A*∪*B *_are the volumes of surface A, B and the superimposed construct AB, respectively. Volumes are calculated using the weighted Delauney triangulation and alpha shape methods [27-29].

The overlap volume is then used to calculate a Tanimoto coefficient, which is a normalized similarity measure[30,31]. By using the self overlap volumes (*V*_*AA*_, *V*_*BB*_), we define the surface volume overlap Tanimoto (SVOT):

$$SVOT_{AB}=\frac{V_{AB}}{V_{AA}+V_{BB}-V_{AB}}$$

A SVOT score is bounded between 0 (representing non-overlapping surfaces) and 1.0 (representing identical surfaces). The SVOT values for the spatial alignment series show in Figure 3d are 0.50, 0.52, 0.73, 0.62, 0.91, and 0.89 when viewed from left (black) to right (white). The best spatial alignment, as judged by RMSD values, does not guarantee the best SVOT score (i.e. SVOT scores are not correlated to RMSD).

**Figure 4:**
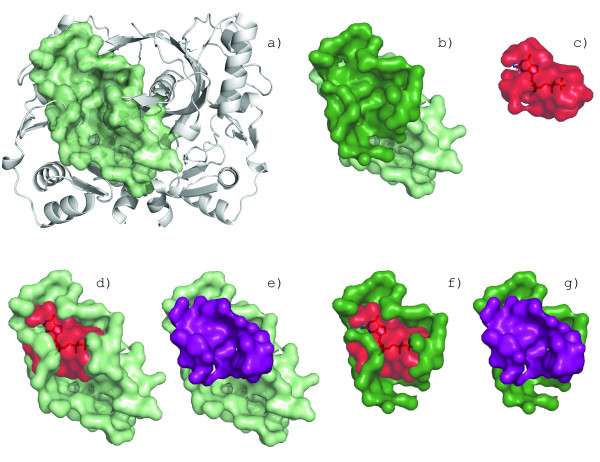
**Calculating volume overlap between aligned surfaces**. A surface on F420-0:gamma-glutamyl ligase homolog from *A. fuldgidus *(PDB:2g9i) (a) has a well conserved sub-surface (b, forest green) to the GDP binding surface in GDP-binding protein from *B. taurus *(c). A superposition of the surfaces from the alignment (d). When the volume overlap of the alignment is measured (e, purple), the large volume disparity between the surfaces masks the similarity with global surface volume overlap (gSVOT) score of 0.37. Using only the conserved residues of the alignment (f) to measure the local global volume overlap (lSVOT) reveals the similarity with lSVOT score of 0.71 (g, purple).

The interpretation of the SVOT score is not straightforward for surfaces that have a large volume disparity. Figure 4 shows a large surface pocket on F420-0:gamma-glutamyl ligase homolog from *A. fuldgidus *(PDB:2g9i, a) where a sub-surface (b, forest green) is highly conserved to the GDP binding surface in GDP-binding protein from *B. taurus *(PDB:1tad, c). The volume overlap of the superimposed surfaces has a calculated SVOT score of 0.37, suggesting low similarity (Figure 4e, purple). In this case, the SVOT score fails to account for strong sub-surface similarity; hidden by the overall volume disparity.

To improve the alignment scoring, we compute two versions of the SVOT, the local and global surface overlap volumes. In the global SVOT (gSVOT), we apply the rotation matrix from the conserved residues to the entire surface (Figure 4e). The local SVOT (lSVOT) is limited to the subset of conserved residues from the alignment solution (Figure 4f). The local and global SVOT are calculated as follows:

$$gSVOT_{AB}=\frac{V_{AB}}{V_{AA}+V_{BB}-V_{AB}},lSVOT_{ab}=\frac{V_{ab}}{V_{aa}+V_{bb}-V_{ab}}$$

Where *V*_*a *_and *V*_*b *_are the volumes of the surfaces formed only by the conserved residues used in the alignment solution. The lSVOT for the alignment in Figure 4g is 0.71, conveying stronger similarity.

Finally, we introduce the ratio SVOT (rSVOT), to establish a correspondence between the global and local surface volumes:

$$rSVOT=\frac{gSVOT_{ab}}{lSVOT_{AB}}$$

The rSVOT is bounded between 0 and 1 and represents the fraction of a surface utilized in the alignment solution. In Figure 4, the rSVOT value of 0.52 confirms that it is a sub-pocket match. The rSVOT score can be used to automatically detect surfaces that do not have the desired properties for a particular search (e.g. excluding sub-surface matches during a library search).

### Automated Identification of Ligand Binding Surfaces

Functional surfaces of proteins can be derived from structural information extracted from three-dimensional coordinates. This task can be automated for PDB files deposited with heteroatom records (HETATM) describing atoms belonging to small molecule cofactors rather than to part of a biopolymer chain. To extract the functional surfaces that surround structural features of a protein, an exclusion contact surface is generated by measuring a difference in solvent accessibility between a structure with and without a molecule in proximity. This is illustrated for the NADP binding surface from human pathogen *S. pyogenes *(PDB:2ahr) in Figure 1a. Atoms with a change in solvent accessibility between the bound and apo structure are identified as the contact surface (Figure 1b). We utilize the Delauney triangulation and alpha shape method for measuring solvent accessibility [27-29].

#### A Library of Protein Functional Surfaces

An exclusion contact surface has been calculated for every heteroatom molecule associated with a protein's PDB file and organized into the Global Protein Surface Survey (GPSS). The GPSS contains three-dimensional libraries of functionally annotated surfaces from ligand, DNA, metal and peptide binding surfaces and is updated weekly to correspond to PDB deposits. Libraries are publicly accessible through a web browser[32] or via a PyMol[33] plugin. In this study, we utilize only the ligand binding surfaces of the GPSS: 113,921 members representing 5,575 unique ligands (PDB version: November 2006). For this subset of the GPSS, the average number of residues forming a surface is 12 and the average molecular weight of the bound ligands is 305. To reduce redundancy and improve search efficiency, we further limit our search library to a single ligand of each type from each protein deposit. The first ligand of each type, as described in the PDB file, was selected.

### Similarity Searching Surface Libraries

**Figure 5:**
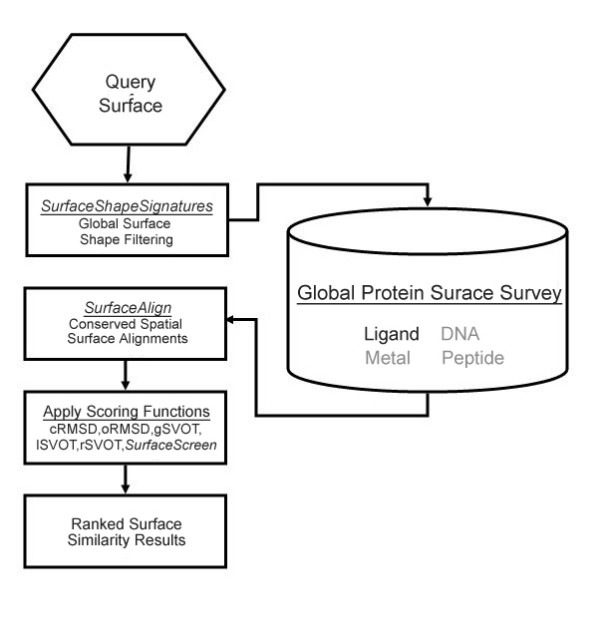
**The *SurfaceScreen *methodology uses the *SSS *algorithm to rapidly pre-classify surfaces based on shape complimentarity**. Similarly shaped surfaces are then spatially aligned using the *SurfaceAlign *algorithm and scored. While the GPSS library also contains surfaces from DNA, metal and peptide binding surfaces, in this study, only ligand binding surfaces were considered.

We incorporate our two comparison algorithms, *SurfaceShapeSignatures *and *SurfaceAlign *into a comprehensive searching methodology, *SurfaceScreen*, to query a protein surface against the GPSS library. The GPSS library contains pre-computed binding surfaces for ligand, DNA, metals, and peptides from all structures in the PDB. *SurfaceScreen i*s outlined in Figure 5. Given a protein structure, we first identify all solvent accessible surfaces on the structure utilizing the CASTp[34] database and select a query surface of interest. The query surface is compared to each member of our surface library using *SSS*. Each surface, whose shape is not within a threshold, is eliminated from the library. In this manner, the *SSS *is used as a fast pre-classifier before the computationally intensive alignment algorithm and scoring functions are applied. Finally, the spatial alignment is performed and the alignments are scored and ranked.

Independently, each scoring function conveys unique properties about a surface alignment, but an overall score is necessary to consistently evaluate and rank surfaces in a database search. To this end, we define the *SurfaceScreen *score:

$$SurfaceScreen\text{ Score}=(1-SSS)+\frac{\log(cRMSD\;P-value)}{-9}+gSVOT$$

The *SurfaceScreen *score is bounded between 0 and 3, and represents contributions from global shape similarity, local spatial residue alignment, and global alignment volume overlap. The denominator in the cRMSD P-value component reflects the maximum probability value estimated by our statistical significance evaluations, 10^-9^. The oRMSD, lSVOT, and rSVOT scores are omitted because they are too highly correlated to the cRMSD and gSVOT scores, respectively. They are, however, still useful for post-processing results.

### Data Analysis and Classification

#### Receiver Operator Characteristic Curves

Surface retrieval benchmarking experiments are summarized in a Receiver Operator Characteristic (ROC) curve, where the sensitivity is plotted against its specificity at various significance levels of summed probabilities. In the ROC curve, the x-axis represents the false positive rate, or 1-specificity, which is calculated by as 1-TN/(TN+FP), where TN is the number of true negatives and FP is the number of false positives. The *y-*axis represents the true positive rate, or sensitivity, and is calculated as TP/(TP+FN), where FN is the number of false negatives. An overall performance measure of a classification test can be calculated by the area under the ROC curve (AUC)[35]. Bound between 0 and 1, an AUC of 1 is indicative of a perfectly accurate classification test, in which all true positives are distinguished from false positive. An AUC of 0.5 corresponds to a random classification test (e.g. a coin flip). The AUC is a combined measure of sensitivity and specificity.

For our *SurfaceScreen *methodology, the ROC curves measure our ability to accurately identify similar surfaces from a large database. A true-positive data point in our database retrieval experiments is defined when the ligand from the query surface matches the ligand from the corresponding library surface (e.g. retrieving heme binding sites when a heme binding site is used as the query). This definition should be considered conservative, as protein surfaces have the ability to bind multiple ligands and, in some cases, a false-positive prediction may indeed be a biologically relevant hit.

#### ATP Conformation Classification

The coordinates for all ATP molecules in the PDB were identified and extracted. Multiple occurrences of the ligand in structure deposit were included and treated as unique molecules. A pairwise, least-squares superposition of all remaining molecules was performed and RMSD values recorded in a distance matrix. Complete linkage clustering was applied to the data matrix. For clarity, we chose to discover the minimum number of conformation families that would accurately represent all ATP molecules. A range of cut values was tested and we chose to set the number of clusters to four based on manual visualization and analysis. The four conformations represent the bent, extended, and two intermediary forms of ATP.

## Results and discussion

### Surface-Based Retrieval of Binding Sites for the Same Protein: HIV-1 Protease

The utility of surface shape comparisons was assessed by retrieving functionally homologous human immunodeficiency virus (HIV-1) protease-ligand complexes from the GPSS. HIV-1 protease is an essential aspartyl protease that cleaves nascent polypeptides enabling maturation of viral proteins. Inactivation of the protease blocks production of infectious viral particles[36]. Therefore, HIV-1 has been an active target and one of the early success stories of rational drug design[37]. We identified 151 HIV-1 protein-inhibitor complexes deposited in the PDB with the following criteria: proteins are in the dimer conformation, inhibitors are not compound fragments (molecular weight >100), and inhibitors are unique in our dataset. The proteins in our dataset share at least 48% sequence similarity and secondary structure similarity Z-scores greater 9.0, as measured using the secondary structure matching (SSM) algorithm[38].

**Figure 6:**
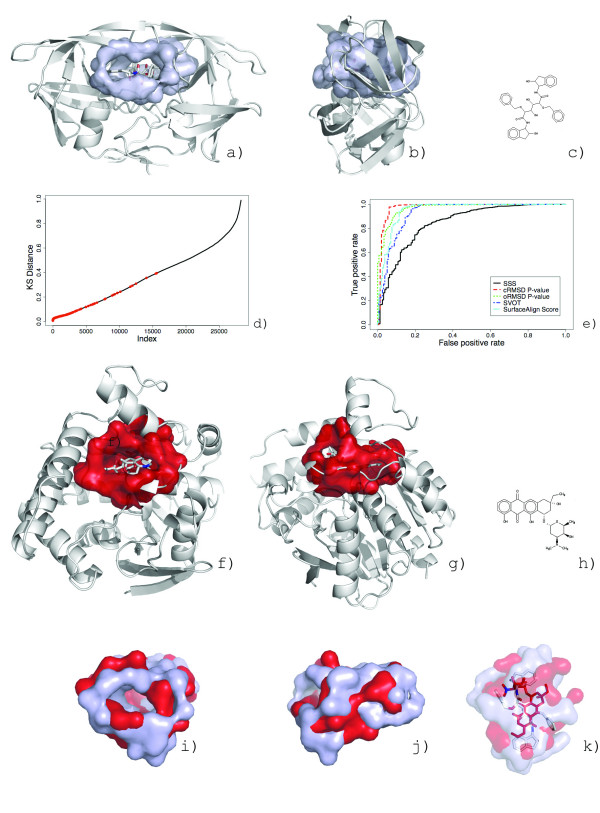
**Retrieval of HIV-1 proteases from the GPSS library using surface similarity**. The binding surface of human HIV-1 protease (ab) complexed with inhibitor BEB (c) was queried against the GPSS library. The sorted KS distances are shown in (d) with other HIV-1 proteases highlighted in red. ROC curves for retrieval using *SurfaceShapeSignature, SurfaceAlign *and *SurfaceScreen *scoring are shown in (e). The highest ranking non-protease surface was from the DcmaT (h) binding surface aclacinomycin methylesterase (RdmC) from *S. purpurascens *(fg). A superposition of the surfaces based on the *SurfaceAlign *alignment (ij) and with their respective ligands (k).

The binding surface of human HIV-1 (PDB:1eby, E.C. 3.4.23.16, CATH[39] 20.40.70.10, Figure 6ab) with bound inhibitor BEB (MW 652.7, Figure 6c) was selected as a query. First, the query was searched against the GPSS library using the SSS comparisons. The sorted KS distance scores between the query surface and all members from the library are plotted in Figure 6d. Points highlighted in red indicate known HIV-1 inhibitor binding surfaces. The results behave expectedly as 124 of 151 have KS distance scores less than 0.1. Plotting the search results in a receiver operator characteristic (ROC) curve (see Methods) we measure the retrieval rate using *SSS *at 84.7% from the area under the curve (AUC) (Figure 6e). The poorest ranking HIV-1 protease surfaces are associated with aggressive mutation studies in the binding pocket or correlated to decisively small (<200) or large (>900) molecular weight inhibitors.

Next, we performed the same search using only the spatial alignment scores to evaluate similarity. We observe that all three alignment-based scoring measures provide better specificity than SSS distance score. The AUC for cRMSD P-value, oRMSD P-value, and SVOT are: 97.5%, 96.8% and 93.0%, respectively. The ROC plots are shown for each measure in Figure 6e. The improved specificity of the spatial alignment scores comes at a significant runtime disadvantage. The SSS shape retrieval method took 24 minutes to compare the query to the GPSSS library, while the spatial alignment took 1,657 minutes. When using the SSS scores to pre-filter the search library, as described in the *SurfaceScreen *methodology, we can achieve an AUC of 95.3% for the combined *SurfaceScreen *(Figure 6e) score with an overall runtime of 148 minutes. The shape signature filter reduced the library 86%, to just over 4,000 surfaces, yet did not eliminate any true positives from the library.

Using the *SurfaceScreen *score, the most similar (rank 132) non HIV-1 surface was from plasmepsin II from *P. falciparum *(PDB:1lf3, E.C. 3.4.23.39, CATH 2.40.70.10), another aspartic protease and a major virulence factor[40].*P. falciparm *is a species of *Plasmodiums *that causes one of the major malaria infections in humans. Plasmepsin II plays an essential role in *P. falciparm *in the degradation of hemoglobin as a source of amino acids for growth and maturation. The binding surface of inhibitor EH5 exhibited strong similarity to the HIV-1 inhibitor binding site, with a *SurfaceScreen *score of 1.89. Plasmepsin II shares only 12% sequence identity to HIV-1 protease and an SSM alignment produces a non-significant Z-score of 3.3. The surfaces are both formed at the intersection of loops and β-sheets, although the plasmepsin II binding surface is formed from a single chain, unlike HIV-1 protease, which is occurs at a dimer interface. There are 15 residues that are conserved between the two surfaces. While it is not surprising that proteases share a similar binding site, the low level of sequence and secondary structure similarity highlights that localized functional conservation can be found in surfaces. Our observation is in agreement with recent reports where HIV-1 inhibitors have been shown to be effective antimalarial agents[41].

The highest-ranking non-protease surface was from aclacinomycin methylesterase (RdmC) from *S. purpurascens *(PDB:1q0r, Figure 6fg). RdmC modifies the aklavinone skeleton in the biosynthesis of anthracyclines in *Streptomyces *species[42]. Anthracyclines are a class of aromatic polyketide antibiotics used as chemotherapuetic agents to treat a wide range of cancers, including leukemia, lymphoma, and breast, uterine, ovarian and lung cancers. Despite sharing only 7% sequence identity and being built present in different scaffolds (CATH 3.40.50.1820), the RdmC binding surface of DcmaT (Figure 6h), was found to be similar with a *SurfaceScreen *score of 1.81 (ranked at position 134). The superposition of the surfaces is shown in Figure 6ij and with their corresponding inhibitors in Figure 6k. The surprising similarity of these surfaces has significant medicinal impact as it supports the recent reports of the inhibitory effects of anthracycline agents on protease activity[43]. This result shows how identification of similar binding surfaces (and their corresponding ligands) can provide guidance for structure based drug discovery, not only in scaffold design but also to screen against potential undesirable binding site similarities that could result in undesired side effects.

### Retrieval and Prediction of Heme Binding Surfaces

**Figure 7:**
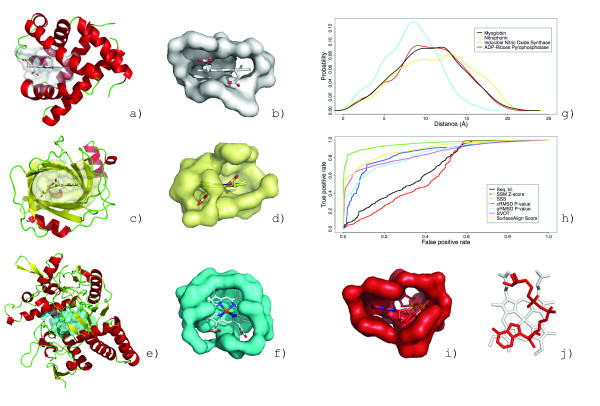
**Retrieval of functionally diverse heme binding proteins**. Heme binding proteins myoglobin (a, CATH code = 1.10.490.10, PDB:1mbn), nitrophorin (c, CATH code = 1.40.128.20, PDB:1np4), and inducible nitric oxide synthase (iNOS) (e, CATH code = 3.90.1230.10, PDB:4nos)[46]. The structures are positioned such that the proprionate groups are all oriented in the same direction. The corresponding heme binding surfaces are shown adjacent, after being rotated 90 degrees along the Y-axis. Shape signatures for each surface are shown in (g). The ROC curves for retrieval of heme binding surfaces querying myoglobin from *P. catodon *(PDB:1mbn) against the GPSS library (h). The Ampcpr binding surface (i) from ADPRase is the best non-heme binding surface returned from the search. A superposition of the ligands suggests ligand-shape complimentarity driving the binding surface similarity (j).

Heme is a versatile prosthetic group that plays an important role across many biological systems. Hemoproteins have diverse functions including oxygen binding and transport, electron transfer and redox, and catalysis. Their functional diversity is accomplished through an equally diverse range of protein topologies[9,44]. A comprehensive analysis of 68 b-type heme binding interactions by Schneider *et. al*. identified over 20 different folds that bind heme in both solvent accessible cavities and buried voids[45]. Even functional homologues show diversity in binding orientation as observed in HasA and HemS[45]. Surfaces from myoglobin (CATH code = 1.10.490.10, PDB:1mbn), nitrophorin (CATH code = 1.40.128.20, PDB:1np4), and inducible nitric oxide synthase (iNOS) (CATH code = 3.90.1230.10, PDB:4nos)[46], representing extrema of heme binding, are shown in Figure 7.

The variability of heme binding proteins presents considerable challenges for automated identification and retrieval of hemoproteins from sequence and structure databases. Using myoglobin from *P. catodon *(PDB:1mbn, Figure 7ab) as a query protein, we compared it to a non-redundant (<95% sequence identity) PDB set using BLAST[47]. Using the 690 heme binding proteins in the PDB as true-positive hits, a retrieval rate of 68.7% is calculated from the rank order of sorted (by E-value) search results. Comparing the structure of myoglobin against the same set of proteins using SSM search results provides a retrieval rate of 64.4%. The retrieval rate was calculated from the rank order of sorted Z-scores. The ROC plots are shown for both methods in Figure 7h.

The myoglobin query was then searched against the GPSS library. The retrieval rate, using the *SurfaceScreen *score, is 94.8%. All surface scoring measures had superior performance over sequence and structure methods (Figure 7h). The most selective was the SSS KS distance with retrieval at 95.8%. Despite the variability of topologies forming binding surfaces, the binding surface shape appears to be the most conserved feature of hemoproteins. This can be seen in the shape signature plots for myoglobin, nitrophorin and iNOS (Figure 7h). The iNOS heme binding pocket is the lowest ranking true-positive surface against our query, as observed by the stark difference in shape signatures. It appears that despite evolutionary pressure imposed for functional specification, surface must maintain geometry necessary to accommodate the canonical heme shape. Surface shape is better conserved for heme binding than the amino acid environment. These observations agree with that of Schneider in which heme binding interactions were found to be generally diverse, with the exception of only three amino acids at "hot spots".

While true heme binding surfaces dominate the top scoring surfaces we find that other binding surfaces have surprising similarity to our query surface. The highest ranking, at position 42, was the Ampcpr binding surface from ADP-ribose pyrophosphatase (ADPRase) from *E. coli *(PDB:1khz). A Nudix hydrolase enzyme, ADPRase catalyzes the Mg^2+^-dependent hydrolysis of ADP-ribose to AMP and ribose 5-phosphate[48]. The surface, formed at the intersection of β-sheets and loops, is shown in Figure 7ij. The SSS plot confirms that strong visual shape similarity between the two surfaces (Figure 7g, red). A shape-based superposition (ROCS, OpenEye Scientific, Inc.) of the ligands shows that (Figure 7j) the similarity of these functionally unrelated proteins may lie strictly in their ability to accommodate similarly sized molecules.

#### Detection of a Convergent Heme Binding Surface

Convergent evolution presents a far more difficult challenge for annotation of proteins of unknown function. Structural genomics target, IsdG from *S. aureus*[49] (PDB:1xbw), shows no significant sequence similarity and does not contain the conserved N-terminal histidine or the GXXXG motif characteristic present in the heme-monooxygenases family, yet this enzyme displays classical heme-monooxygenase activity[49]. Also, while all known members of heme-oxygenase superfamily are of all α-helical fold, IsdG adopts α+β sandwich with an anti-parallel β-sheet and ferredoxin-like fold and a β-barrel at the dimer interface[49].

**Figure 8:**
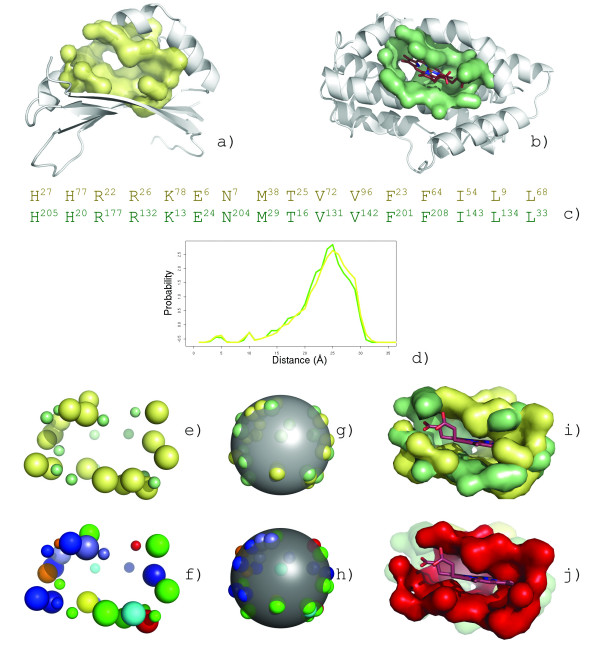
**Identification of a convergent heme binding surfaces from surface similarity**. Despite lacking sequence or structural homology to the heme-monooxygenase family, IsdG from *S. aureus *(a, yellow) contains a conserved surface allowing it to perform heme-monooxygenase activity. When compared to the heme binding surface from heme oxygenase (HmuO) from *C. diphtheria *(b, green), 19 residues are conserved (c) with similar global shape characteristics (d). The superposition of the conserved residues is shown for the best scoring cRMSD (e) and oRMSD (g) alignments. The alignments are colored by residue type (IsgG large radius, HmuO small radius) in (fh). The superposition of the surfaces resulting in the maximum volume overlap (i, red) is shown with bound heme from HmuO (j).

The structure of IsdG has a prominent pocket formed between the α-helices and beta sheets (Figure 8a). This is the largest surface pocket identified by the CASTp webserver[50]. Querying this surface against the GPSS library reveals a striking similarity to the heme binding pocket in heme oxygenase (HmuO) from *C. diphtheriae *(PDB:1iw0, Figure 8b). The SSS distributions have distance of 0.06 (Figure 8d). There are 19 conserved residues between the surfaces that come from diverse regions of the primary sequence (Figure 8c). The surfaces align with cRMSD P-value of 9.84 × 10^-3 ^(Figure 8ef) and oRMSD P-value of 5.32 × 10^-4 ^(Figure 8gh). Superposition of the surfaces results in gSVOT of 0.78, lSVOT 0.84, and rSVOT of 0.93 (Figure 8i). The gSVOT overlap is highlighted in Figure 8j. The *SurfaceScreen *score for the comparison is 1.98, which ranked fourth overall against the search library.

**Figure 9:**
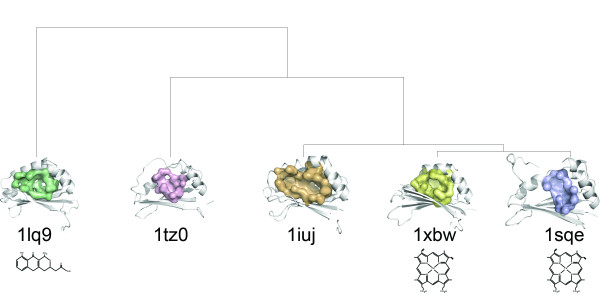
**Binding surface-based classification of structural homologs**. Putative binding surfaces for structural genomics targets with structural homology to IsdG (PDB:1xbw) and IsdI (PDB:1sqe) from *S. aureus *are clustered by *SurfaceScreen *scores. The heme binding pocket is well conserved in protein TT1390 from *T. thermopilus *(PDB:1iuj) and protein BC2969 from *B. cereus *(PDB:1tz0). ActVA-Orf66 from *S. coelicolor *(PDB:1lq9), is known to bind 6-deoxydihydrokalafungin (6-DHHK)[51]. Cofactors are shown immediately below each protein.

Several other structural homologues to IsdG have subsequently been solved in the structural genomics effort. A clustering of the putative binding sites for four additional enzymes is shown in Figure 9. Surface analysis reveals that the heme binding pocket is well conserved in protein TT1390 from *T. thermopilus *(PDB:1iuj) and protein BC2969 from *B. cereus *(PDB:1tz0) suggesting that both *T. thermopilus *and *B. cereus *can acquire iron through heme degradation. Despite overall structural similarity, the binding surface is not well conserved in ActVA-Orf66 from *S. coelicolor *(PDB:1lq9), a protein involved in antibiotic synthesis that is known to bind 6-deoxydihydrokalafungin (6-DHHK)[51]. Expectedly, this surface forms the most distant branch of the clustering. Although all proteins appear to function as monooxygenases they operate on very different substrates suggesting that convergent evolution may be an important driving force to evolve new functions from existing protein scaffolds. In this manner, surface analysis could be used to define a chemical structure space by interpolating between known substrates clustered on each node.

### Binding Site Retrieval of Functionally Diverse and Conformationally Variable Nucleotides

#### Specific Nucleotide Binding Site Retrieval

**Figure 10:**
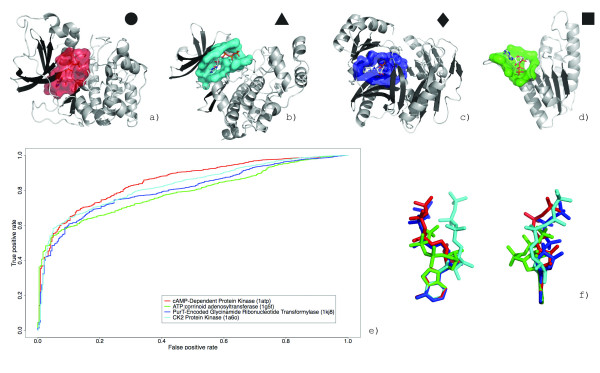
**Retrieval of ATP binding proteins from functionally and conformationally diverse classes**. Binding surfaces representing different ATP conformational classes: cAMP- dependent kinase (PDB:1atp, a), protein kinase CK2 from Z. Mays (PDB:1a6o, b), ATP:corrinoid adenosyltransferase from S. typhimurium (PDB:1g5t, c), PurT-encoded glycinamide ribonucleotide transformylase from E. coli (PDB:1kj8, d). A superposition of the molecules from each class (f). The retrieval rate for each binding surface against the GPSS library is shown as an ROC plot in (e). The retrieval rates are calculated using the *SurfaceScreen *score.

ATP is a multifunctional nucleotide associated that has been classified to catalyze 58 different reactions by the Enzyme Commission (EC). In over 300 structural complexes, ATP binding is associated with domains from 45 homologous superfamilies, some sharing less than 8% sequence identity[52]. The nucleotide is quite flexible and adopts a wide range of conformations, some in less than energetically favorable states[52]. To determine the extent that conformational variability exerts on similarity searching, we conducted retrieval experiments with query surfaces binding ATP in diverse conformations: cAMP- dependent kinase (PDB:1atp) protein kinase CK2 from *Z. mays *(PDB:1a6o)[53], ATP:corrinoid adenosyltransferase from *S. typhimurium *(PDB:1g5t)[54], PurT-encoded glycinamide ribonucleotide transformylase from *E. coli *(PDB:1kj8)[55]. The conformations were selected by clustering all ATP molecules by their three-dimensional shape similarity (see Methods) and are shown in Figure 10.

The AUCs calculated using the *SurfaceScreen *score were 79.1%, 80.1%, 83.0%, and 85.4% for ATP:corrinoid adenosyltransferase, PurT-encoded glycinamide ribonucleotide transformylase, protein kinase CK2 and cAMP-dependent kinase, respectively (Figure 10e). The extended ATP form, which is the most prominent form in the PDB, had the best retrieval rate, while the bent form ATP had the poorest. Overall, the rates underperform compared to the HIV-1 inhibitor and heme binding surface retrievals. Despite the influence of ligand conformation of surface conformations, our method appears rather tolerant to flexible ligands (and their corresponding binding surfaces) albeit at the expense of the specificity seen in more rigid molecules.

It should be noted that the retrieval rates for ATP are especially conservative, as a disproportional number of ATP binding surfaces complexed with other molecules are in the PDB. For example, there are 11 structures of Protein Kinase A from *Bos taurus *(PDB:1xha,1xh8,1xh7,1xh6,1xh5,1xh4,1veb,1svg,1sve,1svh) which have ATP competitive inhibitors bound. By correcting for protein kinase inhibitor complexes, the AUCs improve approximately 4% across all query surfaces. Unfortunately, there is no automated method to associate these types of complexes with their native cofactors except through literature analysis, which can be uninformative, as every structure deposit does not have a corresponding publication. The authors are developing an automated database that catalogs such natural cofactor/inhibitor relationships between structures in the PDB.

#### Non-specific Nucleotide Retrieval

In some proteins, ligand binding is not an exclusive event, as some proteins are able to utilize different cofactors to catalyze the same reaction. Casein kinase 2 (CK2) is a highly conserved eukaryotic serine/threonine kinase that plays a key role in various cellular processes and possesses dual-cosubstrate specificity for guanosine-5'-triphosphate (GTP) or ATP[53]. This feature, whose biological significance is not well understood, is exceptional among eukaryotic protein kinases. Querying the CK2 ATP binding surface (Figure 10b), we can retrieve GTP binding surfaces from the GPSS library with AUC of 83.7%, slightly better than ATP. Given that the two molecules differ only in their nucleoside, this result is not surprising, as we have shown ligand shape complimentarity is a strong precursor of overall surface similarity. In a comparison of the surface retrieval rates for all nucleotides binding surfaces in the PDB against the CK2 ATP binding surface, we observe trends which mirror ligand shape similarity: purine derivatives retrieval is better than the pyrimidines and tri-phosphate molecules retrieval is better than di-phosphates which are retrieved better than mono-phosphates. These results suggest that our method may be useful to identify a diverse set of molecular shapes that could potentially bind to a given surface.

#### Prediction and Validation of a GDP Binding Site

**Figure 11:**
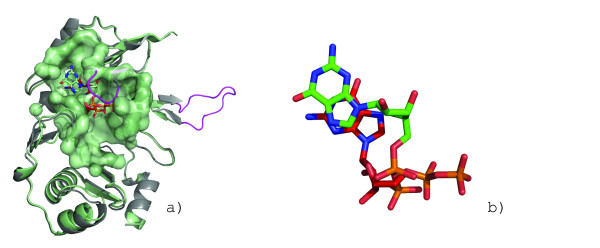
**Crystallographic validation of GDP binding prediction in structural genomics target**. The strong similarity of the putative binding surface of F420-0:gamma-glutamyl ligase homolog from *A. fuldgidus*[56] (a) to the GDP binding surface in GDP-binding protein from *B. taurus *(Figure 3c) allows a GDP molecule (red, colored by element) to be posed into the surface based on the surface superposition. The structure was determined with bound GDP (green, colored by element) with RMSD of 1.0Ǻ from predicted position (b). The addition of the ligand to the crystallization conditions improved the quality of the structure from 2.5Ǻ (a, gray) to 1.35Ǻ (a, green) and allows loop regions (magenta) to be modeled.

The F_420 _coenzyme plays important roles in archaea and eubacteria in a variety of biochemical reactions (e.g. methanogenesis, the formation of secondary metabolites, the degradation of nitroaromatic compounds, DNA repair)[56]. CofE, a F_420_-0:gamma-glutamyl ligase, is responsible for the last two enzymatic steps in coenzyme F_420_-2 biosynthesis. Belonging to a structurally uncharacterized family of enzymes, CofE from *A. fulgidus *was solved as a structural genomics target by the Midwest Center for Structural Genomics and found to be of novel fold (PDB:2g9i) (Figure 4a). Solvent accessible cavities were calculated using the CASTp webserver [27-29,34], and the largest pocket, presumably the F420, GTP and L-glutamate binding pocket, was selected to query against the GPSS ligand surface library. The top-ranking surface was from GDP-binding protein from *B. taurus *(PDB:1tad, red, Figure 4c). A GDP molecule is posed into the surface based on the superposition from the alignment (Figure 11a, red GDP molecule). Based on this prediction, the protein was co-crystallized with GDP and a model of the complex was determined (Figure 11a, green GDP molecule)[56]. The GDP position had RMSD of 1.0Ǻ from the predicted pose (Figure 11b). The addition of the ligand also improved the resolution of the structure from 2.50Ǻ to 1.35Ǻ and allowed two loop regions to be modeled where no electron density was previously seen (Figure 11a, magenta).

### ATP Binding Surface Landscape

**Figure 12:**
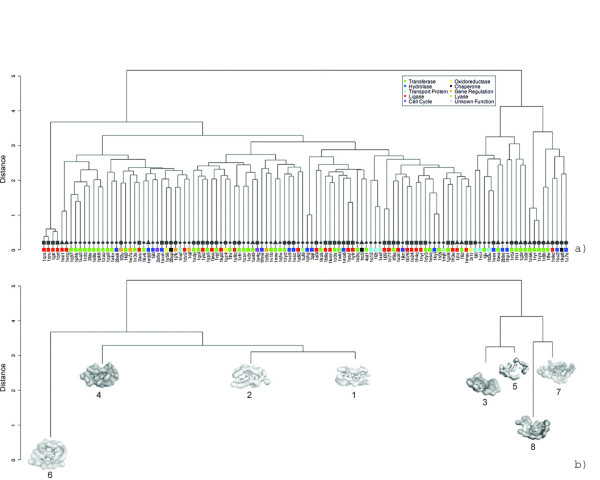
**Clustering of 116 non-redundant ATP binding sites based on their surface similarity**. The dendrogram represents the results of complete-linkage clustering, applied to SurfaceScreen score between all surfaces in our dataset (a). Each node is color-coded representing its biological functions as assessed through EC numbers or literature references. A second grayscale-coded shape can be found on all node edges that corresponds to the ATP conformations in Figure 9. A representative binding surface from each cluster is shown in (b).

The contributions of molecular flexibility only partially account for reduced retrieval rates observed for ATP binding surfaces. It is surprising that binding surfaces for this essential nucleotide do not exhibit a greater level of conservation. To explore the global relationship between surfaces, enzymatic functions and ligand conformation, we carried out an all-against-all comparison for a limited homology dataset (<50% whole sequence identity) of ATP binding surfaces. This cutoff was selected to encourage surface diversity between functionally homologous proteins yet eliminate redundant analysis. A distance matrix for the 116 surface set was constructed using the *SurfaceScreen *score and complete linkage clustering was applied. A dendrogram of the clustering is shown in Figure 12.

**Figure 13:**
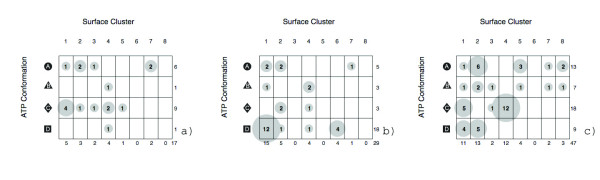
**Mapping ATP binding surface cluster membership and ATP conformation class**. Observed frequencies for hydrolases (a), ligases (b), and transferases (c) are shown. Surface cluster numbers correspond to Figure 11(b). ATP conformation class labels correspond to Figure 9. The sums for each row and column are shown on the edges of each plot.

The cluster results show that there is minimal functional exclusivity between binding surfaces and ATP conformation. The same enzymatic functions can be accomplished using a variety of binding surfaces and, within each surface, multiple ligand conformations can be bound. In the most well represented functional families, hyrdolases, ligases, and transferases, we observe different degrees of binding mode conservation. A breakdown of surface clustering and ligand conformations is shown as a balloon plot in Figure 13. Hydrolases have two conformation preferences and favor, deep, encapsulating binding surfaces. Bent form ATP is disfavored in hydrolases. Ligases are the most conserved, heavily favoring the bent form of ATP that requires a wide-mouth surface shape. Transferases are the most adept of the ATP binding proteins, sampling the most surface/conformation combinations. They do not discriminate between ATP conformations but have a preference for encapsulating binding sites. Several combinations occur with higher frequencies, including an exclusive combination (4-◆), which is the most observed in this family.

Analysis of a broad collection of ATP binding proteins suggest that some functional families may have conserved binding surfaces while others are more divergent. Binding surfaces themselves also deviate on their level of ligand conformation tolerance. It is likely that altering protein surfaces may be the most cost effective evolutionary mechanism for exploiting functional niches, even within functional families.

#### Automated Protein Kinase Classification by ATP Binding Site Comparisons

Within the transferase family, the most well conserved ATP binding surfaces belong to protein kinases. Protein kinases play vital roles in regulating cellular pathways by phosphorylating other proteins. Malfunctioning kinases have been linked to a variety of diseases such as immunodeficiency, endocrine disorders and cancer, making them the target of drug discovery efforts. At their highest level, kinases are divided by the amino acid residues they target (serine/threonine or tyrosine) and further classified by more specific biochemical activity. Functional classification of kinase families has been undertaken by many methodologies utilizing primary sequence, structure, binding sites, pharmacophore profiles and expert manual analysis [57-60]

We apply our comparison methodology to protein kinases to assess our ability to automatically classify them into their functional subfamilies using only ATP binding surfaces. A dataset of 297 protein kinases with bound ligands, including both natural and synthetic molecules, were annotated using a combination of the PDB web query system, EC numbers, KinBase[61] and the Protein Kinase Resource[62]. For consistency, family nomenclature is applied from KinBase.

**Figure 14:**
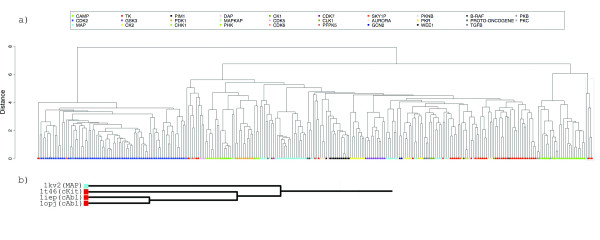
**An all-against-all comparison of ATP binding surfaces in the PDB**. The dendrogram represents the results of complete-linkage clustering, applied to *SurfaceScreen *score between all surfaces in our dataset (a). The nodes of the dendrogram are color coded for kinase families according to KinBase nomenclature. A branch of the cluster (gray box) is called-out to highlight the unexpected similarity discovered between the STI-571 binding site in c-Abl kinase and serine/threonine kinase p38 MAP (b).

An all-against-all comparison was performed with results used to populate a distance matrix of *SurfaceScreen *scores. A dendrogram showing the complete-linkage clustering is shown in Figure 14 where each surface node is color coded by kinase subfamily. Overall, the method shares strong agreement with the annotated classification, as seen by the color banding. CDK2 kinases are the most ordered; with all members perfectly clustered together and distinct sub-grouping separating nucleotide ligands from small compound inhibitors. The CK2 and CAMP families also show divergence between natural ligands and inhibitors. The CAMP groupings are further clustered by the molecular weight of their bound ligands. The mitogen activated protein kinases (MAP) are successfully classified into their sub-families, but on distant nodes in the graph. In all families, we observe differentiation based on the activation state of the kinase.

Surface based classification conveys many similarities to other methods, but has advantages of additional functional insight that could not be automated in other methods. The ability to distinguish between different activation states and to organized surfaces based on ligand types and molecular weight differences could prove useful in developing binding profiles for enhanced specificity in kinase drug discovery.

#### Binding Surface Similarity of c-Abl Kinase Inhibitors

The surreptitious fusion of the cellular form of Abelson leukemia virus tyrosine kinase (c-Abl) with the breakpoint cluster region (BCR) gene disrupts the internal control mechanism causing increased tyrosine kinase activity[63]. The fusion protein BCR-Abl results in the disease chronic myelogenous leukemia (CML). Five structures of c-Abl proteins can be found in the PDB with two classes of small molecule inhibitors: pyrido [2,3.d]pyrimidine-type (PDB:1m52, 1opk) and 2-phenylaminopyrimidine-type (PDB:1iep, 1fpu, 1opj). Both inhibitor classes bind in the ATP binding, but 2-phenylaminopyrimidine-type bind exclusively in the inactive conformation of the activation loop. The smaller pyrido [2,3.d]pyrimidine-type class are indifferent to activation state, making them more potent but less specific inhibitors[64]. Our clustering accounts for this behavior and groups them in distinct nodes.

**Figure 15:**
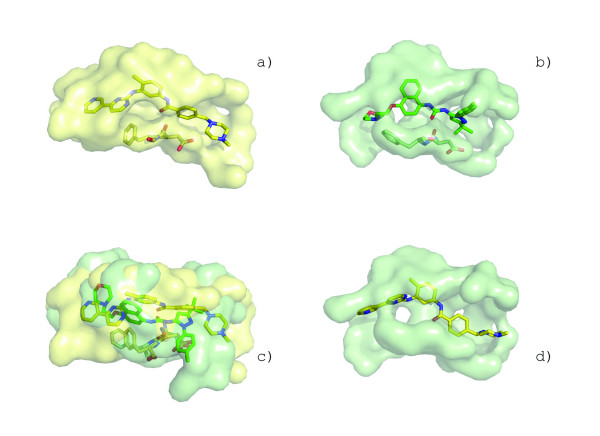
**Unique conformation of p38 MAP kinase creates similar binding surface to c-Abl kinase**. The binding surface of inhibitor STI-571 in c-Abl kinase (a, PDB:1opj) shows strong similarity to the binding surface of inhibitor B96 in p38 MAP kinase (b, PDB:1kv2). p38 MAP kinase has DFG motif configuration (stick representation) similar to that seen in c-Abl. *SurfaceAlign *superposition of the surfaces (c). STI-571 is posed into the p38 MAP binding surface based on the surface alignments (d).

The 2-phenylaminopyrimidine-type inhibitor STI-571 (Figure 15a) is an effective inhibitor of c-Abl activity for treatment against CML [64-66]. It has been shown to be specific for tyrosine kinases and also inhibits stem-cell factor receptor kinase c-Kit (PDB:1t46). Results from querying c-Abl (PDB:1opj, Figure 15a) against the GPSS library show cKit is the best scoring non-ABL kinase. This cross reactivity is detected in our cluster (Figure 14b).

A surprising member of this cluster node is serine/threonine kinase p38 mitogen-activated protein (MAP) kianse (PDB:1kv2, Figure 14b). p38 MAP kinases play critical roles in regulation of proinflammatory cytokines such as tumor necrosis factor and interleukin-1 and are a target for many inflammatory diseases[67]. STI-571 is not currently known to inhibit, by design or mechanism, any serine/threonine kinase[68]. The structure of 1kv2, complexed with inhibitor B96, occupies a unique conformation for p38 MAPs, where the highly conserved DFG motif is turned out[67] (Figure 15b). This is the first observation of this state in a serine/threonine kinase, where it is a hallmark for tyrosine kinases (Figure 15a).

A superposition, based on the alignment of the binding surfaces of c-Abl and p38 MAP, shows that the inhibitors bind in similar orientation (Figure 15c). STI-571 can be posed into the p38 MAP surface, based on the alignment of the surfaces, with no steric clashing and preserves the orientation of several polar atoms (Figure 15d). While the conformation of this p38 MAP is unique and presumed to occur infrequently, its existence presents opportunity to explore the use of STI-571 and analogs for additional therapeutic uses. Automated surface classification can also provides important cross-reactivity analysis; where unexpected binding sites similarities could result in undesired side effects.

## Conclusion

Proteins maintain the surprising ability to preserve local, sequentially unordered, surface residue patterns capable of performing explicit biochemical functions in proteins showing negligible evolutionary relationships. Even in homologous proteins, subtle amino acid mutations, which can be underappreciated by sequence analysis, can alter the properties of a surface and protein function. In this study, we describe a novel method for the comparison and analysis of protein functional surfaces. We observe that conservation of surface shape and physicochemical texture provides sufficient discriminative features for accurate retrieval of functionally homologous binding sites. The method serves as a predictive tool allowing for the identification of cross-binding ligands or binding sites on proteins of unknown function and for the comparative analysis of proteins, such as the classification of functionally diverse families. We introduced the Global Protein Surface Survey, a searchable library of functionally annotated protein sites. The *SurfaceScreen *methodology was benchmarked against binding pockets from HIV-1 protease, heme, and ATP and used to further analyze the relationship between surface similarity, biochemical function and ligand conformation.

### Limitations and Outlook of SurfaceScreen

Our results have shown that two components, shape and physicochemical texture, define well the functional competence of surfaces. Surface geometry allows accessibility and proximity for interaction and accurate residue positioning make available specific functional groups for biochemical function. A notable limitation to our current method is our spatial residue model, which does not afford for amino acid substitutions during spatial alignments. Previous studies have shown that the substitution rates for localized surfaces differ from those of the whole sequences[69,70] and that these differences can provide better discrimination in surface sequence comparisons. One option would be to exclude residues less likely to be involved in function. For example, in the study of general enzyme function, Ysteng *et al*[69] studied 3,275 functional surfaces to discover His, Asp, Glu, Ser, and Cys residues account for more than 80% of active site residues in functional pockets. This is similar to previously published reports [71-73]. Defining a minimum residue set describing different functions is plausible, but would reintroduce difficulties arising from global versus local surfaces characteristics and properties. This would also naively assume that some of the residues excluded in a scheme are not contributing in some way, either mechanistically or structurally, to the function. Many proteins also show high promiscuity and can bind several different ligands into this same functional site[74], solvent mediated interactions add complexity to the surface comparisons[75] and electrostatic potential also plays important role in conformational changes and attracting or rejecting ligands[76]. It is clear that in the future analysis of protein surfaces properties will need to include contribution from electrostatic potential, side chain dynamics, and chemical propensities to better describe functional sites.

## Authors' contributions

AJ and TAB conceived of the study and performed analysis on provided examples. TAB designed the methodologies and implemented the software. All authors read and approved of the final manuscript.
